# Safety and tolerability of pasireotide long-acting release in acromegaly—results from the acromegaly, open-label, multicenter, safety monitoring program for treating patients who have a need to receive medical therapy (ACCESS) study

**DOI:** 10.1007/s12020-016-1182-4

**Published:** 2016-11-28

**Authors:** Maria Fleseriu, Elisha Rusch, Eliza B. Geer, on behalf of the ACCESS Study Investigators

**Affiliations:** 1 Oregon Health & Science University, Portland, OR USA; 2 Novartis Pharmaceuticals Corporation, East Hanover, NJ USA; 3 Division of Endocrinology, Mount Sinai Hospital, New York, NY USA

**Keywords:** Acromegaly, Expanded access trial, Fasting plasma glucose, Glycated hemoglobin, Hyperglycemia, Pasireotide LAR

## Abstract

**Purpose:**

Pasireotide long-acting release is a somatostatin analog that is indicated for treatment of patients with acromegaly. This analysis documents the safety of pasireotide long-acting release in patients with acromegaly enrolled in the ACCESS trial (ClinicalTrials.gov identifier: NCT01995734).

**Methods:**

ACCESS is an open-label, multicenter, single-arm, expanded-treatment protocol designed to provide patients access to pasireotide long-acting release pending regulatory approval. Patients received pasireotide long-acting release 40 mg administered intramuscularly every 28 days. The primary outcome was the proportion of patients having a treatment-emergent grade ≥3 or serious adverse event. Efficacy data were not collected.

**Results:**

Forty-four adult patients with active acromegaly were enrolled in the study for an average of 37.6 weeks (range, 4–70 weeks). Twenty-five grade ≥3 treatment-emergent adverse events were reported in 11 patients (25.0 %), 3 of whom (27.3 %) experienced grade ≥3 hyperglycemia. In patients treated with pasireotide long-acting release for ≥3 months (*n* = 42), mean glycated hemoglobin and fasting plasma glucose levels increased significantly from 5.9 % and 100.4 mg/dL at baseline to 6.8 % and 135.9 mg/dL at 3 months, respectively. Ten patients (22.7 %) were treated with pasireotide long-acting release for ≥15 months, after which mean glycated hemoglobin and fasting plasma glucose levels were 6.3 % and 123 mg/dL, respectively. Twenty-one patients (48 %) initiated antidiabetic medication.

**Conclusions:**

Grade ≥3 adverse events (primary outcome) were reported in 25.0 % of acromegaly patients treated with pasireotide long-acting release in a clinical setting. Hyperglycemia-related adverse events were reported in 45.5 % of patients, but were typically manageable, supporting the role of pasireotide long-acting release as a safe treatment option for acromegaly patients.

## Introduction

Acromegaly is a rare disorder that, in the large majority of cases, is caused by hypersecretion of growth hormone (GH) from a benign somatotroph pituitary adenoma, which leads to elevated insulin-like growth factor 1 (IGF-1) secretion [1–3]. Excessive GH and IGF-1 levels (>1 ng/mL and >age and sex-normalized IGF-1 levels above the upper limit of normal, respectively) are associated with multisystemic comorbidities, including congestive heart failure, sleep apnea, arthritis, impaired glucose tolerance, insulin resistance, and diabetes mellitus, as well as an elevated risk of mortality [1, 2, 4]. Treatment that achieves and maintains biochemical control is associated with the improvement of some key comorbidities and leads to normalization of life expectancy [1, 2, 5]. Additionally, sustained reductions in pituitary tumor volume and signs and symptoms of disease are important goals in the treatment of acromegaly [1, 2]. Transsphenoidal surgery is the first-line treatment for acromegaly [2, 3, 6]. Because approximately 75 % of GH-secreting tumors are macroadenomas that invade the cavernous sinus or extend into the suprasellar region [7], most patients will not achieve complete remission from surgery alone and, therefore, will need pharmacological intervention [2].

Somatostatin receptor types 2 and 5 (sst_2_ and sst_5_) are each expressed in approximately 90 % of GH-secreting pituitary adenomas and have been useful for tumor-targeted pharmacotherapy with first-generation somatostatin analogs [SSAs; octreotide long-acting release (LAR) and lanreotide Autogel (ATG)] [8–13]. Pasireotide LAR is a second-generation, multireceptor-targeted SSA that is approved by the US Food and Drug Administration, the European Medicines Agency, and several South American countries for the treatment of patients with acromegaly who have had an inadequate response to surgery and/or for whom surgery is not an option [14–17]. Compared with octreotide LAR and lanreotide ATG, which bind with highest affinity to sst_2_, pasireotide has higher binding affinity for sst_5_ [14, 18, 19]. In a large, randomized, 12-month, Phase 3 trial (C2305 study) in medically naive patients with acromegaly, pasireotide LAR demonstrated superior biochemical control (composite end point of age-normalized IGF-1 and GH <2.5 ng/mL) in comparison with octreotide LAR (31.3 vs. 19.2 %; *P* = 0.007) [20]. Similar results were obtained in the 12-month crossover extension phase of the C2305 trial, in which 17.3 % (95 % confidence interval, 9.8–27.3) of patients with acromegaly who had not achieved biochemical control with octreotide LAR achieved biochemical control with pasireotide LAR [21]. In a separate, large, randomized, 24-week, Phase 3 trial (C2402 study) in patients with inadequately controlled acromegaly, treatment with two different doses of pasireotide LAR resulted in significantly higher rates of biochemical control than did continued treatment with either octreotide LAR or lanreotide ATG (15 % of patients treated with pasireotide LAR 40 mg and 20 % of those treated with pasireotide LAR 60 mg vs. 0 % of those continuing treatment with octreotide LAR or lanreotide ATG; *P* = 0.0006 for pasireotide LAR 40 mg group, *P* < 0.0001 for pasireotide LAR 60 mg group) [22].

In both of these large Phase 3 trials, treatment with pasireotide LAR was associated with higher rates of hyperglycemia-related adverse events (AEs) than were other SSAs [20, 22]. In the C2305 trial, the rate of hyperglycemia-related AEs was 57.3 % in patients treated with pasireotide LAR and 21.7 % in patients treated with octreotide LAR [20]. Grade 3/4 hyperglycemia-related AEs were reported in 9.0 % of patients treated with pasireotide LAR and in 1.7 % of those treated with octreotide LAR. AEs related to elevations in blood glucose were the most common causes of discontinuation in the pasireotide LAR group. In the C2402 trial, hyperglycemia-related AEs were reported in 67 % of patients treated with pasireotide LAR 40 mg, in 61 % of those treated with pasireotide LAR 60 mg, and in 30 % of those who continued treatment with active control [22]. In those patients with normal glycemic levels at baseline, 30 % of patients treated with pasireotide LAR 40 mg developed hyperglycemia compared with 48 % of patients treated with pasireotide LAR 60 mg and 15 % of patients treated with the active control [23]. In contrast, in patients with hyperglycemia at baseline, 52 % maintained elevated glycemic levels after 24 months of treatment with pasireotide LAR 40 mg, compared with 71 % of patients treated with pasireotide LAR 60 mg and 42 % of patients treated with the active control. Here we present safety data from an expanded-treatment protocol that evaluated pasireotide LAR in a clinical practice-based setting, with a focus on the reported incidence of hyperglycemia-related AEs.

## Materials and methods

### Patients

Patients were eligible for inclusion in the study if they met the following inclusion criteria: age ≥18 years; confirmed diagnosis of acromegaly that was caused by a GH-producing pituitary tumor, with circulating age and sex-adjusted IGF-1 levels >1.3 × the upper limit of normal and random GH concentrations >1 µg/mL within 30 days of screening; not controlled by, not eligible for, or refused pituitary surgery; and had Karnofsky performance status scores of >60. A washout period of ≥8 weeks (days −63 to 0) was required before the screening assessment for patients who were receiving medical therapy for acromegaly.

Key exclusion criteria included concomitant treatment with SSAs, GH-receptor antagonists, or dopamine agonists, unless concomitant treatment was discontinued and the washout period was completed before the screening assessment; compression of the optic chiasm that caused any visual field defect for which surgical intervention was indicated; signs or symptoms of tumor compression for which surgical intervention was required; major surgery/surgical therapy of any type within 4 weeks of screening; radiotherapy of the pituitary gland within 4 weeks before screening or failure to recover from side effects of radiotherapy; history of hypothyroidism that was inadequately treated with thyroid hormone replacement therapy; or diabetes with poorly controlled blood glucose, as evidenced by glycated hemoglobin (HbA_1c_) >8 % at screening.

### Study design

ACCESS was designed as an open-label, uncontrolled, single-arm, multicenter, expanded-treatment protocol to provide access to pasireotide LAR to patients with acromegaly for whom medical therapy was appropriate pending regulatory approval. After a screening period, which may have included a 4–8-week washout period for patients previously treated with medical therapy, eligible patients were administered pasireotide LAR 40 mg intramuscularly starting at visit 2 (baseline/week 0) every 28 days (±2 days) until either a discontinuation occurred or pasireotide LAR became both commercially available and reimbursable. Patients were to be transitioned to commercial pasireotide LAR as quickly as possible (not exceeding 6 months after commercial availability) and to have end-of-treatment evaluations on the day of the final dose of the study. For patients who discontinued treatment, follow-up evaluations were performed 28 days after the last administration of study drug.

At the study investigator’s discretion, the dose of pasireotide LAR could be increased to 60 mg for patients with age and sex-adjusted IGF-1 levels greater than the upper limit of normal after the third injection of pasireotide LAR 40 mg. Stepwise dose reductions from 60 to 40 mg and from 40 to 20 mg were permitted for tolerability (e.g., grade ≥ 3 AE or laboratory change directly attributable to pasireotide LAR).

The study was conducted in accordance with the ethical principles of the Declaration of Helsinki and implemented and reported in accordance with the International Conference on Harmonisation Harmonised Tripartite Guideline for Good Clinical Practice. The study protocol and informed consent forms were approved by the institutional review board, independent ethics committee, and/or research ethics board of each study site. All patients provided written informed consent to participate in the trial (ClinicalTrials.gov identifier: NCT01995734).

### Outcomes

The primary objective was to document the safety of pasireotide LAR in patients with acromegaly by determining the proportion of patients with treatment-emergent grade ≥3 or serious AEs. Efficacy data were not collected centrally for this study. Secondary end points included the incidence of AEs; abnormalities in laboratory, vital signs, or electrocardiographic (ECG) results; and changes from baseline in laboratory values, ECG results, and vital signs. Investigators were responsible for evaluating IGF-1 and GH every 3 months to determine whether dose modifications were needed; however, the data were not collected by the sponsor.

### Treatment assessments

The severity of AEs was described as mild (grade 1), moderate (grade 2), severe (grade 3), or life-threatening (grade 4) according to the Common Terminology Criteria for Adverse Events (CTCAE) [24]. The CTCAE is a set of criteria used for the standardized grading of AEs. Notably, hyperglycemia is defined and graded by the CTCAE according to fasting plasma glucose (FPG) level as follows: grade 1, greater than the upper limit of normal to 160 mg/dL; grade 2, >160–250 mg/dL; grade 3, >250–500 mg/dL; and grade 4, >500 mg/dL. Serious AEs included those that were fatal or life-threatening; resulted in persistent or significant disability; were associated with a congenital anomaly or birth defect; jeopardized the patient; or may have required medical or surgical intervention, required inpatient hospitalization, or prolonged an existing hospitalization. Laboratory values were regularly monitored, including hematologic and blood chemistry parameters (including FPG), liver function parameters, coagulation parameters, HbA_1c_, free thyroxine, thyroid-stimulating hormone, serum cortisol, plasma adrenocorticotropic hormone, and urinary parameters. A standard 12-lead ECG was performed for each patient at screening, at baseline (before and 30 min after the first injection), 21 days after each injection for the first 3 months, and every 3 months thereafter during the study. Gallbladder ultrasonography was performed at baseline, at month 6, and at the end of treatment.

### Statistical analyses

Demographic and other baseline data (including disease characteristics) were summarized for the complete cohort, which included all patients who received ≥1 dose of pasireotide LAR. All safety analyses were based on data from patients who received ≥1 dose of pasireotide LAR and had ≥1 postbaseline safety assessment. No sample size calculation was performed. Primary safety data were analyzed with descriptive statistics. Categorical data are represented as frequencies and percentages. For continuous data, means, standard deviations (SDs), medians, minimums, and maximums were estimated.

## Results

### Patient characteristics

**Fig. 1:**
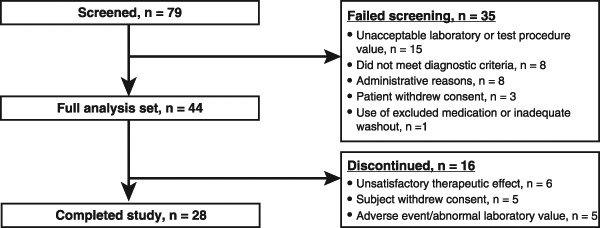
Patient disposition

A total of 79 patients were screened, and 44 were deemed suitable candidates for inclusion in the study (Fig. 1). There were several reasons why patients failed the initial screen, including unacceptable laboratory or test procedure value (*n* = 15; 19.0 %), did not meet diagnostic criteria (*n* = 8; 10.1 %), administrative reasons (*n* = 8; 10.1 %), consent withdrawal (*n* = 3; 3.8 %), and use of excluded medication or inadequate washout (*n* = 1; 1.3 %). For the duration of the study, 44 patients (female, 56.8 %) received treatment for acromegaly with pasireotide LAR. The mean age was 45.5 years, and the mean body mass index was 32.9 kg/m^2^. Previous diagnoses of impaired fasting glucose and diabetes mellitus were reported in 1 patient (2.3 %) and 12 patients (27.3 %), respectively. Patient characteristics at baseline are presented in Table 1.

**Table 1:** Baseline patient characteristics

Characteristics	Patients
	(*n* = 44)
	*n* (%)^a^
Age, mean (SD), y	45.5 (14.5)
Female	25 (56.8)
Race	
White	36 (81.8)
Black	4 (9.1)
Asian	1 (2.3)
Other	3 (6.8)
Karnofsky performance status	
Normal	16 (36.4)
Minor signs or symptoms of disease	15 (34.1)
Normal activity with effort; some signs of disease	8 (18.2)
Cares for self; unable to carry on normal activity	3 (6.8)
Requires occasional assistance but cares for most of own needs	2 (4.5)
Time from diagnosis to first pasireotide dose, mean (SD), mo^b^	63.6 (75.5)
Prior pituitary surgery	36 (81.8)
Time from latest pituitary surgery to first pasireotide dose, mean (SD), mo^c^	62.8 (60.6)
Prior pituitary irradiation	9 (20.5)
Time from latest pituitary irradiation to first pasireotide dose, mean (SD), mo^d^	36.8 (36.7)
Prior medication for acromegaly	33 (75.0)
Prior medication administered within 60 d of study start	11 (33.3)
Time from prior medication to first pasireotide dose, mean (SD), mo^e^	19.7 (34.3)
Prior medication	
Octreotide	20 (60.6)
Lanreotide	10 (30.3)
Pegvisomant	10 (30.3)
Cabergoline	13 (39.4)
Bromocriptine	1 (3.0)
Other	5 (15.2)

*SD* standard deviation.

^a^ Unless otherwise noted

^b^ Forty-three patients were included in the analysis

^c^ Thirty-six patients were included in the analysis

^d^ Nine patients were included in the analysis

^e^ Thirty-three patients were included in the analysis

Not all patients initiated treatment with pasireotide LAR at the same time, and the study had a fixed end point (when pasireotide LAR became commercially available and reimbursable). Accordingly, the total treatment time ranged from 4 to 70 weeks. Patients were treated with pasireotide LAR for a mean duration of 37.6 weeks. A total of 43 patients (98 %) received ≥3 injections of pasireotide LAR, with a mean of 9.8 injections per patient.

A total of 15 patients (34.1 %) had ≥1 dose increase, 4 patients (9.1 %) had a dose reduction, and 17 patients (38.6 %) had a dose delay at some point during the study. The initial dose per injection of pasireotide LAR was 40 mg, and the mean dose per injection over the course of the study was 42.2 mg. Twenty-eight patients (63.6 %) completed the study, while 16 patients (36.4 %) discontinued treatment. Of those who did not complete the study, six patients discontinued because of an unsatisfactory therapeutic effect, five because of withdrawn consent, and five because of an AE. Hyperglycemia was listed as the reason for treatment discontinuation for four patients.

### Adverse events

Regardless of relationship to study drug, a total of 25 grade ≥3 treatment-emergent AEs were reported in 11 patients (25.0 %). The most frequent grade ≥3 treatment-emergent AEs were abdominal pain and elevated blood glucose, which occurred in 3 patients each (6.8 %; Table 2). Of the 25 reported grade ≥3 or serious treatment-emergent AEs, 11 were suspected to be related to pasireotide LAR treatment [elevated blood glucose (*n* = 3), elevated HbA_1c_, type 2 diabetes mellitus, abdominal pain, muscle spasms, alopecia, pancreatitis, pancreatolithiasis, and cholelithiasis] and were reported in 6 patients (13.6 %). One patient who experienced grade 3 abdominal pain subsequently received a diagnosis of grade 4 adenocarcinoma of the colon and discontinued treatment with pasireotide LAR because of treatment of colorectal cancer. The adenocarcinoma of the colon was considered unrelated to treatment with pasireotide LAR. In another patient, a grade 2 adenocarcinoma of the colon was diagnosed only 9 days after administration of the first dose of pasireotide LAR, and the event was considered to be unrelated to pasireotide LAR treatment. During the course of treatment, all 44 patients experienced ≥1 AE of any grade, regardless of relationship to pasireotide LAR (Table 2). A total of 20 patients (45.5 %) experienced hyperglycemia-related AEs. Elevated blood glucose was reported in 10 patients (22.7 %), hyperglycemia in 10 patients (22.7 %), and type 2 diabetes mellitus in 6 patients (13.6 %). Although these three categories of AEs are technically the same, they are reported differently depending on whether the AE was detected as a change in a laboratory value or reported by the clinician. Aside from those related to hyperglycemia, the most common AEs reported were gastrointestinal-related: diarrhea in 17 patients (38.6 %), nausea in 12 patients (27.3 %), and abdominal pain in 8 patients (18.2 %).

**Table 2:** Most common AEs in the ACCESS study (observed with frequency ≥7 %)

	Number of events, *n* (%)			
Treatment-emergent AE^a^	Grade 1	Grade 2	Grade 3	Total (*n* = 44)
Diarrhea	13 (29.5)	4 (9.1)	0	17 (38.6)
Nausea	5 (11.4)	6 (13.6)	1 (2.3)	12 (27.3)
Hyperglycemia^b^	5 (11.4)	5 (11.4)	0	10 (22.7)
Elevated blood glucose^b^	5 (11.4)	2 (4.5)	3 (6.8)	10 (22.7)
Abdominal pain	4 (9.1)	1 (2.3)	3 (6.8)	8 (18.2)
Cholelithiasis	8 (18.2)	0	0	8 (18.2)
Nasopharyngitis	7 (15.9)	0	0	7 (15.9)
Fatigue	4 (9.1)	1 (2.3)	1 (2.3)	6 (13.6)
Hypoglycemia	5 (11.4)	1 (2.3)	0	6 (13.6)
Type 2 diabetes mellitus	4 (9.1)	1 (2.3)	1 (2.3)	6 (13.6)
Vomiting	3 (6.8)	2 (4.5)	1 (2.3)	6 (13.6)
Dizziness	5 (11.4)	0	0	5 (11.4)

*AE* adverse event

^a^ No grade 4 AEs were reported in the below categories

^b^ For technical reasons, AEs related to hyperglycemia were designated as either “elevated blood glucose” if determined only by laboratory values or as “hyperglycemia” if reported by the clinician

### Hyperglycemia and diabetes

**Fig. 2:**
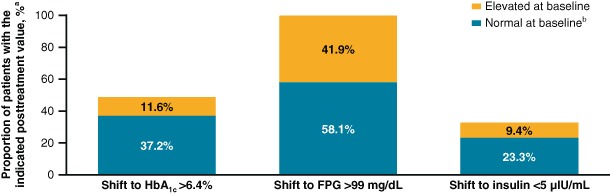
Frequency of patients with ≥1 instance of abnormal glycemic parameters after initiation of pasireotide LAR. **a** Analysis included 43 patients who had evaluable glycemic levels at baseline. **b** Normal ranges: FPG, 70–99 mg/dL; insulin, 5–15 µIU/mL; HbA_1c_, 0–6.4 %. No patients had values below the normal range for HbA_1c_, FPG, or insulin at baseline

Of the 43 patients with evaluable glycemic parameters, elevated levels of HbA_1c_ (>6.4 %) and FPG (>99 mg/dL) were detected at baseline in 5 (11.6 %) and 18 (41.9 %) patients, respectively (Fig. 2). After pasireotide LAR was initiated, HbA_1c_ > 6.4 % and FPG > 99 mg/dL were recorded at least once in 21 (48.8 %) and 43 (100 %) patients, respectively. Low insulin levels (<5 μIU/mL) were detected at least once after pasireotide initiation in 14 patients (32.6 %), whereas no patients had low fasting insulin at baseline.

**Fig. 3:**
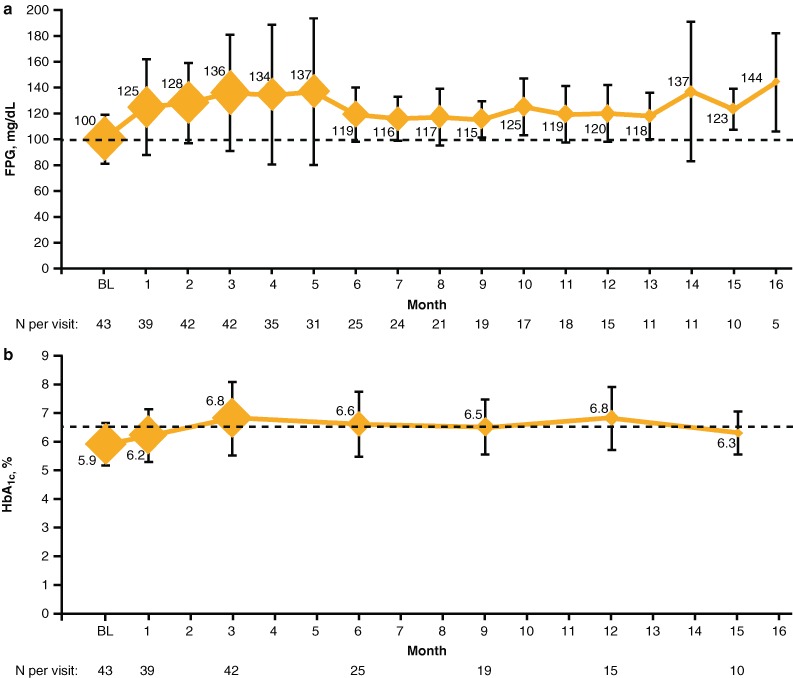
Monthly assessments of **a** FPG and **b** HbA_1c_ during the course of treatment with pasireotide LAR. *Dotted lines* represent the upper limit of the normal range (FPG, 99 mg/dL; HbA_1c_, 6.4 %). *Markers* represent the mean values, and the area of each marker is proportional to the number of patients assessed at each month. *Error bars* represent the standard deviation. Not all patients initiated treatment with pasireotide LAR at the same time, and the study had a fixed end point (when pasireotide LAR became commercially available and reimbursable). Accordingly, fewer patients being evaluated at latter visits occurred primarily because of the different treatment initiation times for individual patients. *BL* baseline

A total of 42 patients (95.5 %) were treated with pasireotide LAR for a minimum of 3 months and had evaluable data for glycemic parameters. Three months after initiation of pasireotide LAR, the mean HbA_1c_ increased from 5.9 % at baseline to 6.8 % (*P* = 0.0002), and FPG increased significantly from 100.4 mg/dL at baseline to 135.9 mg/dL (*P* < 0.0001; Fig. 3). In the 10 patients (22.7 %) treated with pasireotide LAR for 15 months, mean HbA_1c_ levels increased from 5.8 % at baseline to 6.3 % (*P* = 0.1219) and FPG levels increased significantly from 102.4 mg/dL at baseline to 123.2 mg/dL (*P* = 0.0096). Changes in glycemic parameters were accompanied by reductions in fasting insulin levels from a baseline level of 24.5–15.8 μIU/mL at 3 months (*P* = 0.1147). In the 10 patients treated with pasireotide LAR for 15 months, fasting insulin levels were 28.2 μIU/mL at baseline and 19.8 μIU/mL at 15 months (*P* = 0.6079).

Twenty-one patients (47.7 %) initiated new antidiabetic medication on the same day as or after the first dose of pasireotide LAR (Table 3). A range of antidiabetic medications were administered including metformin (*n* = 17), sitagliptin (*n* = 7), any form of insulin (*n* = 3), glimepiride (*n* = 3), linagliptin (*n* = 1), liraglutide (*n* = 1), repaglinide (*n* = 1), PolyGlycopleX^®^ (an over-the-counter supplement, *n* = 1), and canagliflozin (*n* = 1).

**Table 3:** Antidiabetic medications initiated after pasireotide LAR initiation

Antidiabetic medication^a^	Patients
	(*n* = 44)
	*n* (%)
metformin	17 (38.6)
sitagliptin	7 (15.9)
insulin^b^	3 (6.8)
glimepiride	3 (6.8)
linagliptin	1 (2.3)
liraglutide	1 (2.3)
repaglinide	1 (2.3)
PolyGlycopleX^c^	1 (2.3)
canagliflozin	1 (2.3)

*LAR* long-acting release

^a^ instances in which medication was initiated before and continued throughout treatment with pasireotide are excluded. Patients may have taken >1 new antidiabetic medication after pasireotide LAR initiation

^b^ includes any type of fast, intermediate, or long-acting insulin

^c^ PolyGlycopleX is an over-the-counter fiber supplement that is marketed as an aid to help balance blood sugar [25]

## Discussion

Phase 3 studies evaluating the efficacy and safety of pasireotide LAR in patients with acromegaly have demonstrated higher rates of hyperglycemia-related AEs in patients treated with pasireotide LAR than in patients treated with octreotide LAR or lanreotide ATG [20, 22]. The ACCESS study was designed as an expanded-treatment protocol that would permit assessment of the safety of pasireotide LAR in patients with acromegaly within a practice-based setting before the drug became commercially available. The mean duration of treatment was 37.6 weeks, with a mean of 9.8 injections of pasireotide LAR. Our analysis demonstrated a low rate of grade ≥3 or serious treatment-emergent AEs (the primary end point of the study), which occurred in 25.0 % of patients, and only 11.4 % of patients discontinued treatment because of AEs, but 45.5 % experienced hyperglycemia-related AEs. A total of four patients (9.1 %) discontinued treatment with pasireotide LAR because of hyperglycemia-related AEs. Among patients who were treated with antidiabetic therapy, most (66.7 %) initiated only first-line antidiabetic medications (e.g., metformin, sitagliptin). These results suggest that hyperglycemia-related AEs associated with pasireotide LAR may be manageable with standard antidiabetic medication. Of note, <50 % of patients initiated antidiabetic therapy after starting treatment with pasireotide LAR.

In the patients treated with pasireotide LAR for at least 3 months (*n* = 42), elevations in HbA_1c_ and FPG to diabetic levels (HbA_1c_ >6.4 %, FPG >99 mg/dL) were observed in 40 % (*n* = 17) and 93 % (*n* = 39) of patients, respectively, which is consistent with previous findings [20, 22]. Similarly, in patients who completed ≥15 months of the study (*n* = 10), diabetic HbA_1c_ levels (>6.4 %) were reported in 40 % (*n* = 4). Most patients treated with pasireotide LAR, regardless of glucose tolerance at baseline, experience elevated glucose levels within the first 2 to 3 months [14], which typically stabilize thereafter [26]. Therefore, since the majority of patients in the ACCESS study (98 %) received ≥3 doses of pasireotide LAR, the study observation period was likely long enough to allow for appropriate treatment decisions for the management of hyperglycemia related to pasireotide LAR.

Treatment-emergent elevated glucose levels, hyperglycemia, and type 2 diabetes were reported in 23 (*n* = 10), 23 (*n* = 10), and 14 % (*n* = 6) of patients, respectively. However, it should be noted that there is no technical difference between these categories of AEs, except that they are reported differently depending on whether the AE was detected as a change in a laboratory value or was reported by the clinician. To avoid any possibility of inaccurate reporting of the data, the results herein are described exactly as they were recorded in the study (i.e., a distinction was made between “elevated glucose levels” and “hyperglycemia”), similar to the published report of the C2402 study data [22].

The optimal management of hyperglycemia associated with pasireotide LAR in patients with acromegaly is currently being investigated [27, 28]. Of the patients in the ACCESS trial who initiated a new antidiabetic medication, metformin, insulin, glimepiride, and sitagliptin were the drugs most commonly administered. A mechanistic study in healthy volunteers showed that hyperglycemia associated with pasireotide subcutaneous (SC) is associated with decreases in insulin secretion but little change in insulin sensitivity [29]. These findings are consistent with the data presented here, which show a reduction in fasting insulin levels after initiation of pasireotide LAR. In another study of healthy volunteers, treatment with the biguanide metformin was shown to have minimal effects on hyperglycemia associated with pasireotide SC exposure after an oral-glucose-tolerance test, whereas the dipeptidyl peptidase-4 inhibitor vildagliptin and the glucagon-like peptide-1 receptor agonist liraglutide were the most effective at restoring glucose to normal levels [30]. However, a reduction in insulin sensitivity is associated with uncontrolled acromegaly [31]; therefore, it has been suggested that initiating antidiabetic medications that improve insulin sensitivity, such as metformin, may also be an effective strategy for managing pasireotide LAR-associated hyperglycemia in patients with acromegaly [32]. A post hoc analysis of patients enrolled in the C2305 trial showed that mean HbA_1c_ levels after 12 months of treatment with pasireotide LAR were 6.6, 6.7 and 7.2 % in patients whose hyperglycemia was managed with metformin alone, metformin plus other oral antidiabetic medications, and insulin with or without other oral antidiabetic, respectively [27]. These data suggest that metformin may be an effective component in pharmacological control of hyperglycemia associated with pasireotide LAR in selected patients.

This analysis of the ACCESS study provides the first assessment of the safety of pasireotide LAR in a practice-based clinical setting and reflects actual management patterns in a broader population of patients with acromegaly. Limitations of this analysis include an overall small number of patients enrolled in ACCESS who had long durations of time in the trial because of late enrollment in the study, and the lack of specific information regarding doses of concomitant antidiabetic medications.

ACCESS was designed as a safety study, and efficacy data were not recorded as part of the trial design; therefore, correlations between safety and treatment outcomes could not be evaluated. However, previous Phase 3 studies have investigated the efficacy of pasireotide LAR [20, 22]. The study of 358 medically naive patients with acromegaly demonstrated that pasireotide LAR provided biochemical control superior to that of octreotide LAR [20]. Subsequently, a study of 198 patients with inadequately controlled acromegaly showed that treatment with pasireotide LAR resulted in significantly higher rates of biochemical control than did continued treatment with other SSAs [22]. Although efficacy data support the use of pasireotide LAR in the treatment of acromegaly, particularly for patients who fail multimodal treatment, the adverse effects of pasireotide LAR on glycemic levels need to be taken into account when choosing a treatment strategy [33]. The results reported here provide evidence to support the manageable safety profile of pasireotide LAR in patients with acromegaly, which is critical to assessing the overall clinical benefit of treatment with pasireotide LAR.
